# The Opposite Effects of High-Sucrose and High-Fat Diet on Fatty Acid Oxidation and Very Low Density Lipoprotein Secretion in Rat Model of Metabolic Syndrome

**DOI:** 10.1155/2012/757205

**Published:** 2012-10-17

**Authors:** Monika Cahova, Helena Dankova, Eliska Palenickova, Zuzana Papackova, Ludmila Kazdova

**Affiliations:** Department of Metabolism and Diabetes, Institute for Clinical and Experimental Medicine, Videnska 1958/9, Prague 4, 14021 Prague, Czech Republic

## Abstract

*Aims*. To determine the effect of two different diets (high-sucrose (HS) and high-fat (HF)) on the main metabolic pathways potentially contributing to the development of steatosis: (1) activity of the liver lysosomal and heparin-releasable lipases; (2) fatty acid (FFA) oxidation; (3) FFA synthesis *de novo*; (4) VLDL output *in vivo* in a rat model of metabolic syndrome (MetS), hereditary hypertriglyceridemic (HHTg) rats fed HS or HF diets. *Results*. Both diets resulted in triacylglycerol (TAG) accumulation in the liver (HF > HS). The intracellular TAG lipolysis by lysosomal lipase was increased in both groups and positively correlated with the liver TAG content. Diet type significantly affected partitioning of intracellular TAG-derived fatty acids among FFA-utilizing metabolic pathways as HS feeding accentuated VLDL secretion and downregulated FFA oxidation while the HF diet had an entirely opposite effect. FFA *de novo* synthesis from glucose was significantly enhanced in the HS group (fed ≫ fasted) while being completely eradicated in the HF group. *Conclusions*. We found that in rats prone to the development of MetS associated diseases dietary-induced steatosis is not simply a result of impaired TAG degradation but that it depends on other mechanisms (elevated FFA synthesis or attenuated VLDL secretion) that are specific according to diet composition.

## 1. Introduction

Metabolic syndrome (MetS) also known as insulin resistance syndrome is characterized as a combination of cardiometabolic risk determinants including insulin resistance, glucose intolerance, dyslipidemia, nonalcoholic fatty liver disease, and hypertension [1] and is associated with a significantly increased probability of type 2 diabetes development [2]. The liver is partially susceptible to ectopic fat accumulation, one of the most important causal components of MetS, and nonalcoholic fatty liver disease (NAFLD) is now considered to be the hepatic manifestation of MetS.

Hepatic steatosis arises from imbalance in TAG acquisition and removal. The conventional explanation of hepatic triglyceride accumulation is that obesity and insulin resistance result in an increased release of FFAs from adipocytes. Increased adipocyte mass and increased hydrolysis of triglycerides through enhanced activity of a hormone-sensitive lipase contributes to elevated plasma levels of FFAs. Up to date no specific regulation of FFA transport into hepatocytes has been described and hence it is supposed that the rate of hepatic FFA uptake is gun-regulated and therefore directly proportional to plasma FFA concentrations. Nevertheless detailed studies performed by Kalopissis and her coworkers showed that in fat-fed rats the cellular uptake of ^14^C-oleate by hepatocytes *in vitro *is decreased despite significant TAG accumulation in the liver. Qualitatively this phenomenon was observed on different metabolic backgrounds (Wistar, Zucker lean, Zucker obese) and differs only in the extent of its manifestation [3–5]. These observations indicate that the regulation of liver triacylglycerol content is not merely a function of plasma FFA delivery alone but that other intrahepatic mechanisms (i.e., regulation of intracellular TAG breakdown, partitioning of the FFA between oxidation and esterification, *de novo* fatty acid synthesis, regulation of TAG secretion) determine steatosis development. 

Without any doubt dietary factors are one of the significant contributors to the NAFLD phenotype and dietary recommendations are a significant tool in current trends in health promotion. From this point of view a detailed understanding of the impact of different diets on the network of metabolic pathways involved in liver TAG metabolism is essentially necessary. Special attention should be given to the interaction of dietary composition with particular genetic/metabolic background.

Hereditary hypertriglyceridemic (HHTg) rats that were the subject of our study display a majority of the MetS symptoms including hypertriglyceridemia, impaired glucose tolerance, hyperinsulinemia, insulin resistance, and increased blood pressure (see Supplementary Material S1 available at doi:10.1155/2012/757205). This phenotype is manifested even without nutritional stimuli but high sucrose feeding aggravates these symptoms further [6]. The aim of the present study was to determine the effect of two different diets (high-sucrose and high-fat) on the main metabolic pathways potentially contributing to the development of steatosis specifically on genetic background that is particularly prone to the onset of diabetes symptoms. We focused on following metabolic processes: (1) mechanisms regulating intracellular TAG degradation in the liver specifically on the activities of liver lysosomal (LIPA; EC3.1.1.13) and heparin-releasable (HL; EC 3.1.1.3) lipases; (2) partitioning of the released FFA between oxidation and secretion as TAG; (3) on the FFA *de novo* synthesis.

## 2. Materials and Methods

### 2.1. Animals and Experimental Protocol

Male hereditary hypertriglyceridemic rats (HHTg) were kept in a temperature-controlled room at a 12:12-h light-dark cycle, the dark phase from 6 pm till 6 am Animals had free access to drinking water and diet unless stated otherwise. The strain of HHTg rats was originally selected from Wistar strain rats in our laboratory [7]. All experiments were performed in agreement with the Animal Protection Law of the Czech Republic 311/1997 which is in compliance with European Community Council recommendations for the use of laboratory animals 86/609/ECC and were approved by the ethical committee of the Institute for Clinical and Experimental Medicine. 

Starting at 3 months of age (b. wt. 281 ± 3 g), all animals were fed either a high sucrose diet (HS: 70 cal% as sucrose; 20 cal% as protein, 10 cal % as carbohydrate), a high-fat diet (HF: 70 cal% as saturated fat, 20 cal% as protein, 10 cal % as carbohydrate) or a standard laboratory chow diet (SD) for 4 weeks. All diets were isocaloric. (see Supplementary Material S2). Groups designed as “fed” had free access to the diet until termination and the groups designed as “fasted” were food deprived for the last 24 hours.

### 2.2. **Determination of Fatty Acid Synthesis De Novo from Glucose in Liver Slices *In Vitro***

The fasted or fed rats were euthanized between 9–10 am and their liver slices (approx. 1  mm thickness, 150 ± 25 mg) were rapidly dissected. The tissues were incubated for 2 hours in a Krebs-Ringer bicarbonate buffer supplemented with 5 mmol/L unlabelled “cold” glucose, D-[U-^14^C-] glucose (specific activity 20 *μ*Ci/mmol) and 2% bovine serum albumin, gaseous phase 95% O_2_ and 5% CO_2_. All the incubations were carried out at 37°C in sealed vials using a shaking water bath. The estimation of the ^14^C-glucose incorporation into total lipid content was carried out as described previously [8]. Briefly, liver slices were removed from incubation medium, rinsed in physiological solution, and immediately put into CH_3_Cl. The pieces of tissue were dissolved using a teflon pestle homogeniser, methanol was added (CH_3_Cl : methanol 2 : 1) and lipids were extracted at 4°C overnight according to Folch et al. [9]. Next day the residual tissue was removed and the clear extract was taken for further analysis. An aliquot was evaporated, reconstituted in scintillation liquid and its radioactivity was measured by scintillation counting. 

To determine the site (glycerol versus acyl moiety) of glucose incorporated into neutral lipids, an aliquot of clear extract was evaporated and saponified in ethanolic 15% potassium hydroxide at 70°C. Saponification was terminated by adding 5.4 M H_2_SO_4_. After cooling to room temperature the released fatty acids were extracted repeatedly into petroleum ether. The pooled petroleum ether fractions were evaporated, reconstituted in scintillation liquid and the radioactivity was measured by scintillation counting. The amount of radioactivity incorporated into the glycerol residue was calculated as the difference of total activity incorporated into neutral lipids and the petroleum ether fraction of the same aliquot.

### 2.3. **Determination of the Metabolism of Intracellular TAG-Derived Fatty Acid in Liver Slices *In Vitro***

The labelling of cytoplasmic TAG *in vivo* was performed as described by Francone et al. [10]. The rats received an intravenous injection of 20 *μ*Ci ^14^C-palmitic acid complexed to 4% albumin under light ether anaesthesia. The animals were euthanised 90 min later and the preparation of liver slices was carried out as described above. For determination of ^14^C- palmitic acid oxidation to CO_2,_ the experiment was carried out in glass vials with central wells. The vials were capped with rubber stoppers and the reaction was terminated by addition of 0.5 mL of 0.5 M H_2_SO_4_ whereas strips of filter paper soaked with hyamine hydroxide were added to the central wells for collection of ^14^CO_2_. TCA (tricarboxylic acid cycle) intermediate content was measured in the incubated liver slices after homogenisation by UltraTurax (IKA Worke, Staufen, Germany) in 150 mM NaCl. The homogenate was extracted into petroleum ether and radioactivity remaining in the water fraction was counted by scintillation counting. According to Kawamura and Kishimoto [11] this fraction represents mostly TCA cycle intermediates (>80%) and a minor part are amino acids derived from FFA via the TCA cycle (<20%). The ^14^C-palmitic acid incorporation into secreted TAG was determined in a chlorophorm extract of the incubation medium. The conversion of ^14^C-palmitic acid into secreted water-soluble oxidation products (i.e., predominantly ketone bodies) was assessed according to the radioactivity remaining in the aqueous fraction of the incubation medium following the chlorophorm extraction.

### 2.4. Lipase Assay: Exogenous Substrate

Lipase activities were determined in 2% homogenates or subcellular fractions prepared from fresh tissue. 0.5 g of liver was homogenised in 2.5 mL of homogenisation buffer (0.25 M sucrose; 0.001 M EDTA pH = 7.4; heparin 7 IU/mL) on a teflon pestle homogeniser. The homogenate was passed through a nylon mesh, centrifuged briefly in order to remove crude impurities (800 g, 5 min, 4°C) and kept on ice until lipase assay was performed (max. 1 hour). The subcellular fractions were obtained by centrifugation at 10 000 g for 15 min.

The reaction medium for acid or alkaline lipase assay was prepared identically except for the buffer used. ^3^H triolein in toluen was added to 88 mg (100 *μ*M) of cold triolein and the solvent was evaporated under a nitrogen stream. 3% FFA free BSA was dissolved in 4 mL 0.1 M buffer (acetate buffer pH = 4.5 for lysosomal lipase or glycine buffer pH = 9.5 for hepatic lipase) with 150 mM NaCl and 0.05% Triton X-100. The whole mixture was emulsified on a Hielsler sonicator UP200S, amplitude 1 on an ice/water bath 3 min continuously. 40 *μ*L of the homogenate or subcellular fractions were incubated for 60 min at 30°C with the 160 *μ*L of reaction medium in a shaking water bath. After termination of the reaction, the released fatty acids were extracted according to Belfrage and Vaughan [12] and counted for radioactivity.

### 2.5. Lipase Assay: Endogenous Substrate

This approach takes advantage of the coordinated changes in the intracellular localisation of lysosomal lipase and its intracellular substrate. The liver homogenate and subcellular fractions were prepared as described above under iso-osmotic conditions that prevent lysosome disruption. The lysis of lysosomes was induced only after fraction separations during the assay. 20% homogenate was mixed 1 : 1 with 0.2 M acetate buffer pH = 4.5 and incubated for 60 min at 30°C in a shaking water bath. The reaction mixture was extracted into chloroform-methanol and phases were separated by 1 M NaCl. An aliquot of the chlorophorm phase was evaporated and 100 *μ*L of Krebs-Ringer phosphate buffer (pH = 7.6) containing 6% FFA-free BSA was added. The tubes were incubated in a shaking incubator at 37°C for 2 hours. FFA concentration in final KRF/BSA solution was measured using commercially available kit. In order to check the efficiency of FFA solubilisation the emptied tubes were washed with fresh KRB + 6% BSA and then 100 *μ*L of chlorophorm was added. An aliquot was separated by TLC but no substantial traces of FFA were detected.

### 2.6. Determination of TAG Entry Rate into Plasma

The TAG entry rate into circulation was estimated according to Otway and Robinson [13]. Briefly, rats were starved for 24 hrs and then given 1 mL of 10% Triton WR-1339 in 0.9% NaCl or 0.9% NaCl alone intravenously via the tail vein under the light ether anaesthesia. Triton WR-1339 is an alkaryl polyether anionic detergent that blocks the removal of intravascular d < 1.006 g/mL lipoproteins. The animals were sacrificed 90 min after receiving either Triton or 0.9% NaCl by exsanguination via aortic puncture. It has been demonstrated that the concentration of triglyceride in plasma is linear up to 3 hrs after the intravenous injection of WR 1339 hence the time point 90 min after application lies within the linear range. Triglyceride entry (secretion) rate into the plasma was calculated from the following formula: 

TAG entry rate (*μ*mol. 100 g b. wt.^−1^. hr^−1^) = [(T_90_ − T_0_)/1.5] × V × (W/100), where T_0_ is plasma TAG concentration (*μ*mol/mL) before Triton administration, T_90_ is plasma TAG concentration (*μ*mol/mL) at the end of the study, V is plasma volume (mL) and W is body weight. Plasma volume was determined as 3.86 mL/100 g body wt [14].

### 2.7. Triglyceride Content in Tissues

Lipids were extracted from 1 g of fresh tissue homogenised in 1 mL of H_2_O. 0.2 mL of the homogenate was extracted in 15 mL of 2 : 1 chloroform: methanol for 24 hours. The organic and aqueous phases were separated by adding of 6 mL KH_2_PO_4_ and centrifugation at 3000 rpm for 20 min. 1 mL of the organic phase was completely dried, resuspended in 100 *μ*L of isopropylalcohol and 10 *μ*L were used for the analyses. The triglyceride concentration in this aliquot was determined using a commercially available kit (Pliva-Lachema Diagnostics, Czech Republic).

### 2.8. Biochemical Analyses

Nonesterified fatty acids, insulin, triglyceride and glucose serum content and *β*-hydroxybutyrare were determined using commercially available kits (FFA: FFA half microtest, Roche Diagnostics, GmbH Germany; triglycerides: Pliva-Lachema Diagnostics, Czech Republic; glucose: Pliva-Lachema Diagnostics, Czech Republic; insulin: Mercodia, Sweden; *β*-hydroxybutyrate: RanBut, RANDOX, UK).

### 2.9. Chemicals

All materials were reagent grade. ^14^C-palmitic acid and ^3^H-trioleinwere purchased from Amersham, D-[U-^14^C-] glucose was purchased from UVVVR, Prague. FFA free bovine serum albumin (fraction V) was purchased from Serva, palmitic acid and triolein from Fluka, all other chemicals were purchased from Sigma Czech Republic.

### 2.10. Statistical Analyses

Data are presented as mean ± SEM of multiple determinations. Statistical analyses were performed using ANOVA and the Tukey-Kramer multiple comparisons test (*n* = 5–7). Differences were considered statistically significant at the level of *P* < 0.05. Pearson's correlation coefficients were calculated to assess possible relationships between lipase activities and liver triglyceride content and lipase activities and ketone bodies production. 

## 3. Results

### 3.1. Characteristics of Experimental Groups

**Figure 1:**
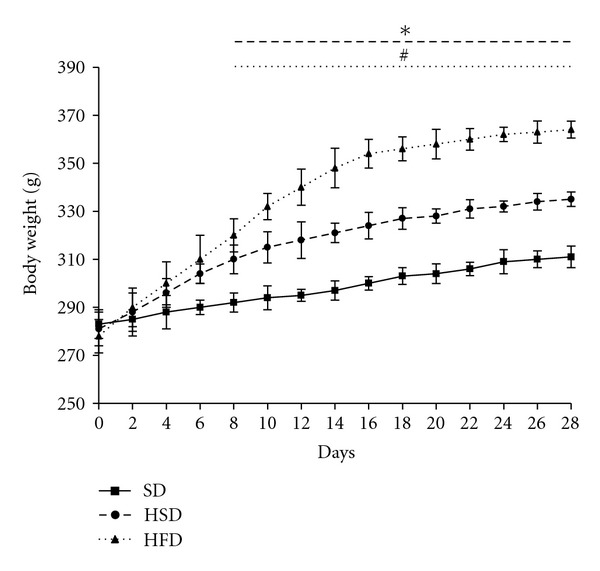
Evolution of body weight in SD, HSD, or HFD fed rats. Body weight was measured three times a week from the beginning of the feeding period till the end of week 4. Mean values ± s.e. obtained in each group are represented. *Significant difference between SD and HSD with *P* < 0.05 or more; ^#^Significant difference between SD and HSD with *P* < 0.05 or less.

The weight of the animals on HS and HF diet (HF > HS) rose rapidly during the first two weeks, then the rate of the weight gain significantly decreased. All tested diets were isocaloric and the more rapid increase in body weight in the HF and HS groups reflects the higher food intake during the first two weeks of diet administration (see Supplementary Material S3). In animals on standard diet this parameter was even throughout the whole experiment (Figure 1). During the last two weeks of diet administration the food intake and the rate of the weight gain in all three groups was comparable. Both final body weight and the weight of epididymal fat pads was higher in HS and HF diet fed animals in comparison with the SD group (HF > HS > SD) (Table 1). The fasting glycaemia and insulinemia were increased only in HF group. The effect of HS and HF diets on serum triacylglycerol levels was the opposite–HS diet significantly increased both fasted and fed triglyceridemia compared to the standard diet while HF diets has slight hypolipidemic effect (fed s-TAG decreased by 30%, *P* < 0.05). The changes in serum FFA content followed a similar trend. Both diets significantly increased the ketogenesis in fasting (HF > HS) but only the HF diet led to the increased production of ketone bodies in a fed state. The hepatic triacylglycerol content in fasting animals was increased by 105% and by 280% after HS and HF feeding, respectively. On standard diet, the liver triacylglycerol content was the same in the fed and fasted states but in the HS group liver TAG content in fed state was significantly lower than in fasting. In contrast, in the HF group the trend was the opposite, hepatic triacylglycerol content being actually higher in fasted than fed animals (Table 2).

### 3.2. **FFA Synthesis *De Novo***

**Figure 2:**
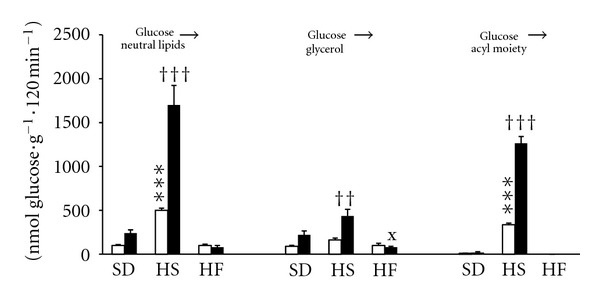
Glucose incorporation into lipids in liver slices *in vitro*. The utilisation of glucose for esterification and *de novo* fatty acid synthesis was determined in the same sample as glucose incorporation into total lipids (described in Section 2). open bars = fasted animals; closed bars = fed animals. All data are means SEM, *n* = 6 individual incubations for each bar. ***P < 0.001 HS-fasted versus SD-fasted; ^††^ *P* < 0.01, ^†††^ *P* < 0.001 HS-fed versus SD-fed; ^x^ *P* < 0.05 HF-fed versus SD-fed.

The liver of rats fed both HS and HF diets contained more triglyceride compared to the SD group. In order to assess the contribution of *de novo *FFA synthesis from glucose to the elevated TAG content, we incubated liver slices prepared from the liver of rats fed each particular diet in the presence of ^14^C-labeled glucose without exogenous FFA and measured the incorporation of glucose into total lipids, into the glycerol part of TAG molecules (i.e., glucose esterification) and into the acyl moiety of TAG molecule (i.e., FFA synthesis *de novo*). As shown in Figure 2, FFA *de novo* synthesis was significantly enhanced in the HS group (fed ≫ fasted) while being completely eradicated in the HF group. 

### 3.3. **The Utilisation of Intracellular TAG-Derived Fatty Acids in Liver Slices *In Vitro***

In order to estimate the accessibility of FFA derived from intracellular liver TAG for further metabolic utilisation we prelabelled cytosolic TAG by the injection ^14^C-palmitic acid *in vivo *and measured the radioactivity incorporation into TCA intermediates, CO_2_ and ketone bodies (FFA oxidation) and into TAG secreted into medium (VLDL secretion) in liver slices *in vitro* 90 min later. As reported by Francone et al. [15], nearly 90% of the radioactive label is found in cytosolic TAG 90 min after the radioactivity administration into the venous blood, so we expect that under this experimental setting most of the FFA incorporated into oxidation products or VLDL had to be released from intracellular TAG by lipolysis. In SD fed animals, both oxidation and VLDL production was lower in liver slices prepared from fed animals compared with those from the fasted ones. The administration of HSD resulted in a significant attenuation of TCA intermediates and CO_2_ production but the fasting ketogenesis was somewhat accentuated in this group (*P* < 0.05) compared to SD fed animals. The TAG secretion was significantly higher in HS fed rats than in the two other groups and the prandial dependent regulation was eradicated. In liver slices from the HFD group, we did not find any effect of the diet on TCA intermediates and CO_2_ production but the ketone body production was significantly elevated in both fasting and fed animals (*P* < 0.001). In contrast, TAG secretion into medium was significantly decreased (*P* < 0.001). Taken together, this data indicates that the diet type does not profoundly affect the total availability of FFA for metabolic utilisation but that it rather influences their partitioning between oxidation and VLDL secretion (Table 3).

### 3.4. The Activity of Alkaline and Acid Lipase

**Figure 3:**
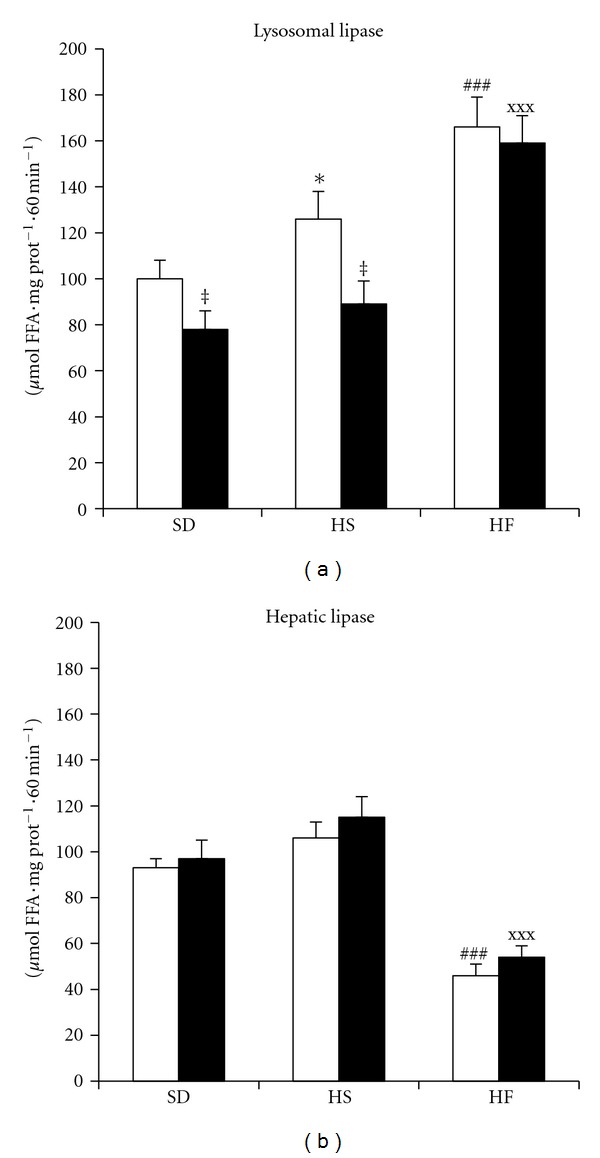
The activity of lysosomal (a) and hepatic (b) lipase in liver homogenate measured as FFA release from artificial substrate (^3^H-triolein). The lipase activity was measured as the release of fatty acids at pH = 4.5 from ^3^H triolein. Open bars = fasted animals; closed bars = fed animals. ^‡^ *P* < fed versus fasted; *P < 0.05 HS-fasted versus SD-fasted; ^###^ *P* < 0.001 HF-fasted versus SD-fasted; ^xxx^ *P* < 0.001 HF-fed versus SD-fed.

As demonstrated above, intracellular TAG's were degraded *in vivo* in the liver of both HS and HF administered animals. In order to analyse the effect of the diets on liver lipolysis in more detail, we directly measured the activity of two liver lipases on exogenous substrate. In liver homogenate, we were able to prove two distinct lipases with optimum pH 4.5 and 8-9. The acid lipase can be identified as “lysosomal” (EC 3.1.1.13) while the alkaline is termed “hepatic” (EC 3.1.1.3). These two enzymes differ in their intracellular distribution and regulation and are affected differently by the administered diets. The effect of the diets on lipase activities in homogenate is shown in Figures 3(a) (lysomal) and 3(b) (hepatic). On standard diet, the activity of lysosomal lipase is higher in fasting and depressed postprandially, whereas the hepatic lipase activity did not differ between fed and fasted animals. Both diets increased the activity of lysosomal lipase but by different amount. On the HS diet, the lysosomal lipase activity increased on fasting but remained unchanged in the fed state. The stimulatory effect of the HF diet on lysosomal lipase activity was significantly higher and was found both in the fasting and fed states. The regulation due to fasting was preserved in rats fed the HS diet but it was completely eradicated in the HF group. The hepatic lipase responded to the tested dietary manipulations differently. HS diet increased the activity of this enzyme both in fasting and postprandially while the HF diet led to significant decrease of its activity.

Centrifugation of liver homogenate for 10 000 g allowed us to separate fractions containing dense lysosomes (pellet) and a fraction containing cytosol, total membranes and light lysosomes (supernatant). On the standard diet, most of the lysosomal lipase activity (85%) was localised in the 10 000 g pellet while most of the hepatic lipase activity (fasting 70%; fed 77%) was found in the 10 000 g supernatant. Neither the HS nor HF diet influenced the subcellular distribution of hepatic lipase, approx. 75% of the activity was found in the 10 000 g supernatant in all experimental settings. As far as the lysosomal lipase is concerned, the HF diet redistributed a significant portion of its activity into the 10 000 g supernatant (Table 4). This data indicate that lysosomal lipase is significantly affected by the HF diet both with regards to the total activity as well as the intracellular localisation. 

**Figure 4:**
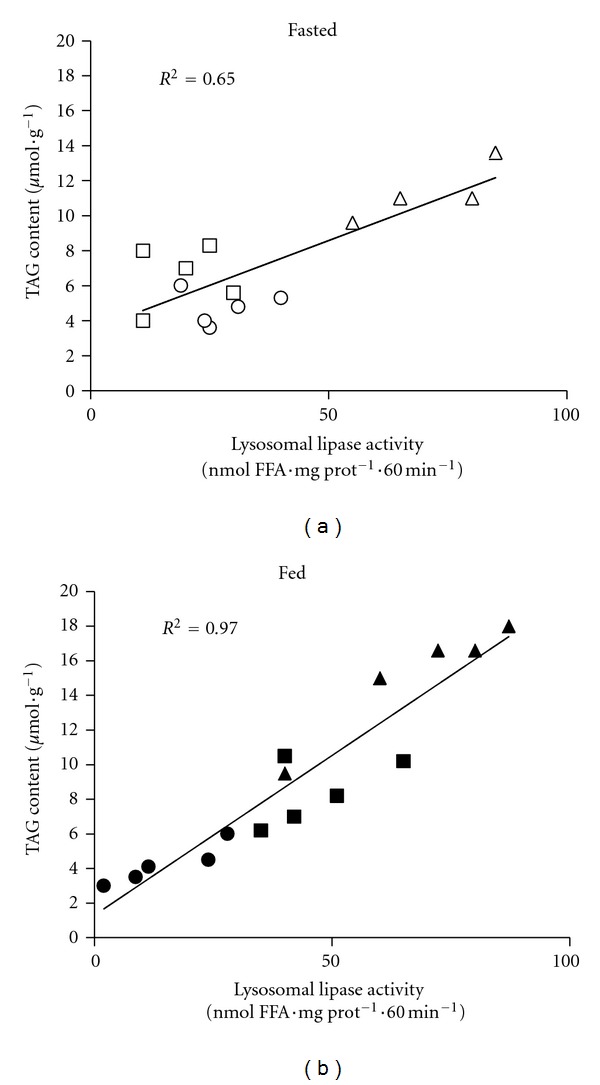
The correlation between lysosomal lipase activity and hepatic triacylglycerol content in fasted (a) and fed (b) animals. Lysosomal lipase activity was determined as the FFA release from endogenous TAG. Open circle = SD-fasted; open square = HS-fasted; open triangle = HF-fasted; closed circle = SD-fed; closed square = HS-fed; closed triangle = HF-fed.

**Figure 5:**
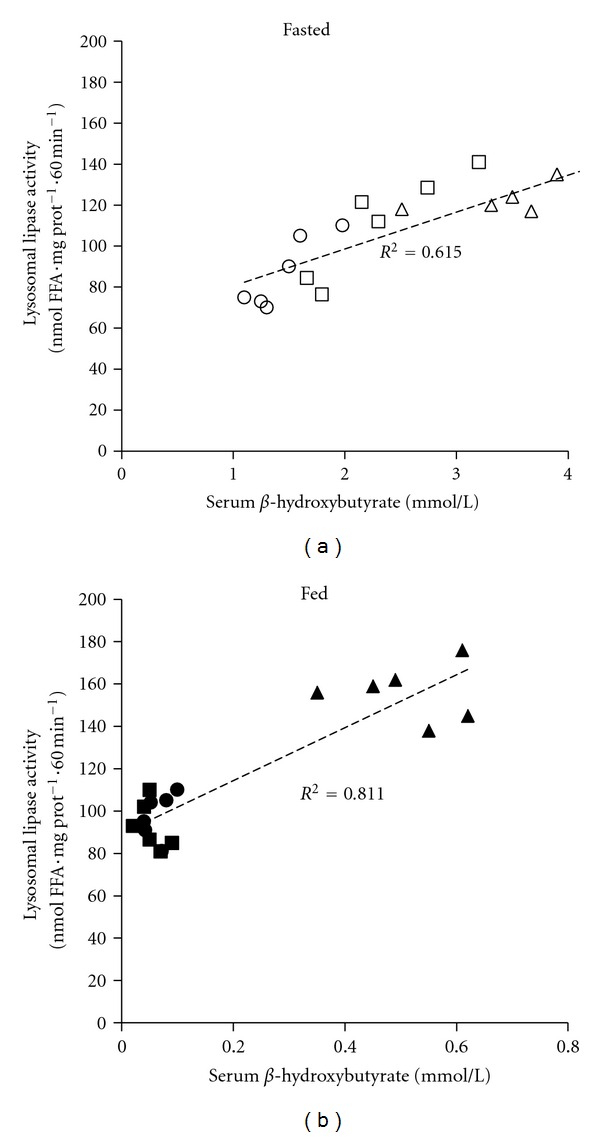
The correlation between lysosomal lipase activity and *β*-hydroxybutyrate content in serum. Lysosomal lipase activity was determined as the FFA release from endogenous TAG. Open circle = SD-fasted; open square = HS-fasted; open triangle = HF-fasted; closed circle = SD-fed; closed square = HS-fed; closed triangle = HF-fed.

The activity of lysosomal lipase determined on endogenous substrate* in vitro* was positively correlated with hepatic triacylglycerol content. This correlation was stronger in the fed (*r* ^2^ = 0.97) than in the fasting state (*r* ^2^ = 0.65) (Figure 4). We found no significant correlation between the hepatic triacylglycerols and hepatic lipase activity (fed: (*r* ^2^ = − 0.4; fasting: (*r* ^2^ = − 0.35) (data not shown). Furthermore the lysosomal lipase is activity positively correlated with serum *β*-hydroxybutyrate concentration both in fasting (*r* ^2^ = 0.81) and fed (*r* ^2^ = 0.62) states (Figure 5). These positive correlations in the case of lysosomal lipase suggest that this enzyme is affected by the availability of the intracellular substrate and that FFA's released by lysosomal lipase action are available at least for ketogenesis.

### 3.5. VLDL Production

The rate of VLDL production *in vivo* was estimated using Triton WR-1339. This detergent effectively blocks the lipoprotein clearance from the blood due to its inhibitory effect on LPL and thus it seems that under such circumstances the accumulation of TAG in plasma could be a valid measure of the rate of VLDL secretion from the liver. As shown in Table 5, TAG entry rate into the circulation is strongly accentuated in the HS group while being significantly diminished in animals administered with HF diet.

## 4. Discussion

The aim of the present study was to contribute to the understanding of the role of different metabolic pathways in the development of hepatic steatosis induced by two different dietary manipulations (HS and HF diet) in the model of the metabolic syndrome. The diet rich in simple carbohydrates or in fat rapidly promotes the TAG accumulation in the liver. It has been suggested that the development of steatosis is associated with the attenuation of intracellular TAG hydrolysis. In contrast to this hypothesis our data indicate that lipolysis, the first step in the intracellular TAG utilisation, is not negatively affected by HS or HF diets. We showed that the important enzyme involved in the intracellular TAG degradation is lysosomal lipase. Furthermore we provided evidence that the mechanism underlying the dietary induced hepatic steatosis depends on the prevailing component of the diet. The HS diet induced steatosis is associated with a significant stimulation of FFA synthesis *de novo*, decreased FFA oxidation and with enhanced VLDL output from the liver. In contrast HF diet associated steatosis is characteristic by downregulated FFA synthesis *de novo,* increased FFA oxidation and significantly impaired VLDL output. 

In spite of decades devoted to the study of TAG metabolism in the liver consensus is still not reached in identification of the exact contribution of particular lipases to the intracellular TAG degradation. In our study, we focused on two lipases present in the liver-heparin-releasable hepatic lipase with pH optimum 8-9 and acid lysosomal lipase with pH optimum 4.5. The activity of hepatic lipase in liver homogenate was increased by HS diet but depressed by HF feeding. Hepatic lipase seems to be induced by chronic hyperinsulinemia [16] which is associated with high sucrose as well. Numerous studies indicate that the expression and activity of hepatic lipase is increased in an insulin resistant state, mostly accompanied by visceral obesity [17]. Nevertheless, most of the activities attributed to the hepatic lipase are oriented out of the hepatocyte, that is, facilitation of the interaction of LDL and remnant chylomicrons with an LRP receptor or participation in HDL metabolism [18–21]. Furthermore, we have previously reported that alkaline lipase is not able to degrade endogenous TAG in the liver homogenate and the only lipolytic activity towards intracellular substrate was found in the acidic range [22]. In perspective of these findings, hepatic lipase is probably not involved in the mobilisation of endogenous triacylglycerols.

Lysosomal lipase, first described by Vavřínková and Mosinger [23], is localised in lysosomes and its activity is elevated in fasting under physiological conditions [24]. In our experiments, this pattern of regulation was preserved only in HHTg rats on standard and HS diets but it was completely eradicated by HF feeding. Both HF and HS diets had a stimulatory effect on the activity of lysosomal lipase but the effect of HF diet was significantly higher than the effect of the diet rich in sucrose. HF feeding led to the translocation of part of the lysosomal lipase activity to supernatant (up to 25%) at the expense of the activity found in pellets. This observation is in accordance with the recently proposed mechanism of lysosome-dependent TAG degradation in the hepatocytes [25, 26]. According to this hypothesis, lipid droplets are incorporated into the autophago(lipo)lysosomes by the process of autophagy, similarly to the autophagy of cytosolic proteins or damaged organelles. The translocation of lysosomal activity from the 10 000 g pellet to supernatant may thus reflect the formation of less dense autophagolipolysosmes containing TAG [27]. We further found a strong positive correlation between acid lipase activity and TAG content in the liver homogenate. Taken together, these data indicate that TAG degradation is actually enhanced in steatosis and that it is mediated by lysosomal lipase. 

In contrast to previously published hypotheses, our results based on direct measurements of acid lipase activity indicate that accumulation of intrahepatic lipids is not the consequence of the impaired mobilisation of intracellular triacylglycerol stores. Despite this, we found positive correlation between the lysosomal lipase activity and oxidation of endogenous triglycerides (ketogenesis) even in the HF fed group which implies that the stored triglycerides are accessible at least for some metabolic pathways. As the capacity of hepatocytes to breakdown intracellular triglycerides is actually raised in both diet groups another factor must explain the different effects of HS and HF diets on triacylglycerol accumulation in the liver and circulation. 

Extensive literature concerning the effect of diet composition (carbohydrate versus fat) on liver TAG metabolism is available. There is a fair consensus that a diet rich in carbohydrates and low in fat resultsin elevation of serum TAG levels, increased VLDL production rate, elevation of TAG amount per particle and attenuation of carbohydrate/fatty acid oxidation [28–32]. However the sensitivity to high carbohydrate intake is highly variable. Numerous studies were performed in order to identify subject characteristics that may be useful in predicting sensitivity to carbohydrate feeding. Characteristics such as sex, TAG concentration when on HF diet, BMI, and insulin concentration have been variably shown to individually predict the effect of the diet and no single variable has a significant predictive value.In contrast, a highly significant effect of the type of carbohydrate has been demonstrated, the effect of monosacharides being much higher in comparison to polysaccharides, and this effect was similar in both hyper- and normotriglyceridemic subjects [33]. Previous studies performed in our laboratory showed that HHTg rats are significantly more sensitive to a HS diet but that the effects imposed by this diet on the TAG metabolism are principally similar in HHTg and their normotriglyceridemic controls although they differ in their magnitude [34, 35]. 

The data concerning the effect of high fat dieton liver TAG metabolism, particularly on VLDL secretion, are more diverse. Several detailed studies focused on this issue and performed on lean and obese Zucker rats were published by Kalopissis group in the 90's. They demonstrated on primary hepatocytes that previous HF feeding (60 cal% as fat, lard) decreases the uptake and utilization of exogenous fatty acids and profoundly influences the intracellular partitioning of FFA released from intracellular TAG in favor of FFA oxidation at the expense of VLDL secretion (40–50% decrease) and lipogenesis (80% inhibition) [3–5, 10]. Of note, qualitatively the effect of HFD on liver TAG metabolism was the same both in lean and obese Zucker rats, but the degree of the changes were more pronounced in the latter. 

In spite of the fact that the HF diet-induced inhibition of VLDL secretion has been repeatedly observed the cause of this phenomenon has not been fully elucidated yet. The availability of glucose is severely lowered in HF diet compared to the standard and HS diets. One of the direct effects of glucose at the hepatic level is the increased secretion of VLDL triacylglycerols [36, 37]. Brown et al. [38] in their study on primary hepatocytes showed that glucose mediated effects are numerous and involve enhanced transport of triacylglycerols out of the cells as VLDL together with an increase in the net synthesis of apoB-48 in the intestine and apoB-100 in the liver. Based on this data, we could speculate that the limiting condition determining the lipid accumulation in liver is the rate of lipoprotein output from the liver which depends on the availability of glucose. Nevertheless, we found a slight increase in fasting serum glucose in HF fed rats which indicates the increased endogenous glucose production. Whether this glucose is available for VLDL production still remains an open question. 

In the presented study we focused on the mechanisms underlying the development of hepatic steatosis in a model that is particularly prone to the dietary manipulation. In conclusion, we provided evidence that in the HHTg rat model of metabolic syndrome hepatic steatosis (both HS- or HF-induced) is not associated with the impairment of intracellular TAG lipolysis. On the contrary, the lysosomal TAG breakdown was actually enhanced under these conditions. Although the effect of both tested diets was qualitatively, but not quantitatively, the same—TAG accumulation in the liver—the underlying mechanisms were different. High sucrose diet was associated with the depression of fatty acid oxidation in parallel with increased TAG secretion and *de novo* FFA synthesis. In contrast in HF diet administered animals the intracellular TAG-derived FFA were channelled predominantly to the oxidative utilisation, namely, ketogenesis, at the expense of a secretory pathway. This finding stresses the importance of understanding exact mechanisms responsible for particular cases in order to choose an efficient therapeutic approach.

## Supplementary Material

Table S1: Characteristics of HHTg rat strain. If not stated otherwise the data were obtained on 3-4 month old animals fed standard diet. Outbred Wistar-Kyoto rats (progenitor strain) were used as the reference. HSD = high sucrose diet.
Table S2: Composition of diets.
Figure S3: Estimation of food intake.

## Figures and Tables

**Table 1:** Characteristics of experimental groups.

		Prediet values	SD (post-diet)	HS (post-diet)	HF (post-diet)	P HS versus SD	P HF versus SD
Body weight (g)		281 ± 3	311 ± 4***	335 ± 15***	364 ± 10***	<0.05	<0.001
Epididymal fat (g)		—	2.8 ± 0.2	3.8 ± 0.3*	5.5 ± 0.5***	<0.05	<0.001
s-glucose (mmol/L)	Fasted	4.5 ± 0.2	4.6 ± 0.1	4.5 ± 0.05	5.4 ± 0.05*	N.S.	<0.05
	Fed	6.2 ± 0.2	6 ± 0.1	11.4 ± 0.1***	7.6 ± 0.4*	<0.001	<0.05
s-insulin (pmol/L)	Fasted	126 ± 18	135 ± 21	155 ± 12	204 ± 20*	N.S.	<0.05
	Fed	155 ± 20	158 ± 15	342 ± 29***	127 ± 18	<0.001	N.S.
s-TAG (mmol/L)	Fasted	1.3 ± 0.25	1.5 ± 0.3	4.9 ± 0.5***	1.4 ± 0.3	<0.001	N.S.
	Fed	2 ± 0.4	2.4 ± 0.2	7.2 ± 0.4***	1.8 ± 0.2	<0.001	<0.05
s-FFA (mmol/L)	Fasted	0.65 ± 0.03	0.7 ± 0.05	1 ± 0.09*	0.6 ± 0.08	<0.05	N.S.
	Fed	0.38 ± 0.02	0.4 ± 0.02	1.3 ± 0.1***	0.45 ± 0.07	<0.001	N.S.
s-*β* hydroxyl butyrate (*μ*mol/L)	Fasted	1.2 ± 0.04	1.3 ± 0.05	2.2 ± 0.14**	3.8 ± 0.2***	<0.01	<0.001
	Fed	0.02 ± 0.01	0.02 ± 0.01	0.03 ± 0.02	0.45 ± 0.05***	N.S.	<0.001

Data are given as mean ± SEM, *n* = 6. *P < 0.05, **P < 0.01, ***P < 0.001 before versus after diet.

**Table 2:** Liver triacylglycerol content.

Diet	Fasted	Fed
SD	3.7 ± 0.3	3.7 ± 0.2
HS	7.6 ± 0.6**	4.8 ± 0.2^†^
HF	14 ± 1.1^###^	20.8 ± 2.3^xxx^

Data are given in *μ*mol·g^−1^ wet weight and expressed as mean ± SEM, *n* = 6.

**P < 0.01 HS fasted versus SD fasted; ^†^ *P* < 0.05 HS fed versus SD fed; ^###^ *P* < 0.001 HF fasted versus SD fasted; ^xxx^ *P* < 0.001 HF fed versus SD fed.

**Table 3:** The incorporation of intracellular TAG-derived ^14^C palmitic acid into CO_2_, TCA intermediates, secreted *β*-hydroxybutyrate, and secreted triacylglycerols from liver slices *in vitro*.

	SD		HS		HF	
	Fasted	Fed	Fasted	Fed	Fasted	Fed
CO_2_ (released)	16 ± 1.2	11.9 ± 1	11 ± 1.5*	8.3 ± 0.3^†^	15 ± 0.7	9.8 ± 0.8
TCA intermediates (intracellular)	55.3 ± 1.6	41 ± 3.4	46.6 ± 1.8**	25.7 ± 1.5^†††^	54.2 ± 4.2	40.3 ± 1
*β*-hydroxybutyrate (secreted)	269 ± 9	204 ± 8	380 ± 18**	178 ± 15	452 ± 22^###^	480 ± 11^xxx^
TAG (secreted)	581 ± 35	401 ± 44	713 ± 29**	825 ± 29^†††^	314 ± 16^###^	286 ± 35^xx^

Data are expressed in nmol palmitic acid per g tissue and given as mean ± SEM *n* = 6. *P < 0.05, **P < 0.01 HS fasted versus SD fasted; ^†^ *P* < 0.05, ^†††^ *P* < 0.001 HS fed versus SD fed; ^###^ *P* < 0.001 HF fasted versus SD fasted; ^xx^ *P* < 0.01, ^xxx^ *P* < 0.001 HF fed versus SD fed.

**Table 4:** The intracellular distribution of lipases activities in liver.

	Lysosomal lipase				Hepatic lipase			
Diet	10 000 g sediment		10 000 g supernatant		10 000 g sediment		10 000 g supernatant	
	Fasted	Fed	Fasted	Fed	Fasted	Fed	Fasted	Fed
SD	86 ± 9.2	85 ± 2	14 ± 1.5	15 ± 2	30 ± 4	23 ± 2	70 ± 5	77 ± 9.5
HS	87 ± 6.8	86 ± 7.3	13 ± 0.8	14 ± 3.6	40 ± 8.3	38 ± 7.3	60 ± 8.2	72 ± 5
HF	82 ± 4	75 ± 3^xx^	18 ± 2^#^	25 ± 2^xx^	38 ± 5.2	24 ± 3.1	62 ± 4.5	76 ± 7

Data are given in % of the sum of the activities in 10 000 g sediment and supernatant prepared from liver homogenates, *n* = 6. ^#^ *P* < 0.05 HF fasted versus SD fasted; ^xx^ *P* < 0.01 HF fed versus SD fed.

**Table 5:** Effect of HS and HF diet on TAG entry rate.

	SD	HS	HF
TAG_0_ (*μ*mol/mL)	3.9 ± 0.2	6.4 ± 0.5^+++^	3.0 ± 0.1^••,¶¶¶^
TAG_90_ (*μ*mol/mL)	7.4 ± 0.4	11.7 ± 0.75^+++^	5.2 ± 0.44^••,¶¶¶^
TAG entry rate (*μ*mol·60 min^−1^·kg)	11.2 ± 0.7	45.7 ± 6.1^+++^	8.2 ± 0.5^••,¶¶¶^

TAG_0_: triacylglycerol serum concentration before WR 1339 administration; TAG_90_: triacylglycerol serum concentration 90 min after WR 1339 administration. TAG entry rate was calculated according to the formula TAG entry rate (*μ*mol·100 g b. wt.^−1^·hr^−1^) = [(T_90_−T_0_)/1.5] × V × (W/100). Data are given as mean ± S.E.M. *n* = 6. ^+++^ *P* < 0.001 HS versus SD; ^###^ *P* < 0.001 HF fasted versus SD fasted; ^••^ *P* < 0.01 HF versus SD; ^¶¶¶^ *P* < 0.001 HF versus HS.
