# Supplementary material for: The Opposite Effects of High-Sucrose and High-Fat Diet on Fatty Acid Oxidation and Very Low Density Lipoprotein Secretion in Rat Model of Metabolic Syndrome
Source: J Nutr Metab. 2012 Oct 17;2012:757205. doi: 10.1155/2012/757205 (PMC3483727; doi:10.1155/2012/757205)
Supplement: Supplementary file 1 — Table S1: Characteristics of HHTg rat strain. If not stated otherwise the data were obtained on 3-4 month old animals fed standard diet. Outbred Wistar-Kyoto rats (progenitor strain) were used as the reference. HSD = high sucrose diet. Table S2: Composition of diets. Figure S3: Estimation of food intake. [file 757205.f1.docx]

**Suppl. 1** *Characteristics of HHTg rat strain*

|  | HHTg vs outbred WKY | note | reference |
| --- | --- | --- | --- |
| body weight | moderately lower |  | Stolba *et al.* 1992  Sebokova *et al.* 1995a |
| s-glucose (fasted) | no difference |  | Stolba *et al.* 1992  Sebokova *et al.* 1995a |
| s-glucose (postprandial) | elevated | significantly elevated after HSD | Cahova *et al.*2004  Stolba *et al.* 1992 |
| s-TAG (postprandial) | elevated | significantly elevated after HSD | Klimes *et al.* 1995 |
| s-FFA (fasted) | elevated | significantly elevated after HSD | Klimes *et al.* 1995 |
| s-cholesterol | no difference |  | Sebokova *et al.* 1993 |
| liver lipogenesis | elevated |  | Vrana *et al.* 1990 |
| secretion rate of TAG | no difference | post-Triton serum TAG after fasting | Vrana *et al.* 1990 |
| LDL receptor activity | no difference |  | Sebokova *et al.* 1993 |
| glucose tolerance | impaired | determined as OGTT | Stolba *et al.* 1992 |
| *in vivo* insulin sensitivity | GIR decreased by 30% | euglycemic-hyperinsulinemic clamp | Stolba *et al.* 1993 |
| glucose metabolic index | decreased in skeletal muscle and WAT |  | Stolba *et al.* 1992 |
| GLUT4 content in skeletal muscle | decreased by 50% |  | Sebokova *et al.* 1995a, Sebokova *et al.* 1995b |
| insulin binding - adipocytes | reduced by 20% | further suppressed by additional 60% after HSD | Fickova *et al.* 1990 |
| insulin binding - hepatocytes | no difference |  | Klimes *et al.* 1990 |
| membrane lipid composition | no difference |  | Sebokova *et al.* 1993 Fickova *et al.* 1992 |
| liver plasma membrane fluidity | no difference |  | Fickova *et al.* 1992 |
| autophosphorylation of insulin receptor | no difference |  | Fickova *et al.* 1992 |
| exogenous basal or insulin-stimulated tyrosine activity | no difference |  | Fickova *et al.* 1992 |
| blood pressure | elevated by 20 mm Hg | further exacerbated by HSD | Stolba *et al.* 1992 |
| plasma catecholamines | elevated |  | Stolba *et al.* 1992  Lichardus *et al.* 1993 |
| vascular reactivity | lower reactivity of aorta to noradrenalin |  | Edelstein *et al.* 1993 |
| red cell ion transport | increased oubain-resistant Na^+^ transport |  | Kunes *et al.* 1994 |

*Characteristics of HHTg rat strain.* If not stated otherwise the data were obtained on 3-4 month old animals fed standard diet. Outbred Wistar-Kyoto rats (progenitor strain) were used as the reference. HSD = high sucrose diet.

**References:**

Cahova M, Vavrinkova H, Meschisvilli E, Markova I, Kazdova L [The impaired response of non-obese hereditary hypertriglyceridemic rats to glucose load is associated with low glucose storage in energy reserves.](http://www.ncbi.nlm.nih.gov/pubmed/15578328) Exp Clin Endocrinol Diabetes. 2004, 112(10):549-55.

Edelstein S, Kyselovic J, Klimes I, Seböková E, Kovácsová B, Kristek F, Mitková A, Vrána A, Svec P. [Effects of marine fish oil on blood pressure and vascular reactivity in the hereditary hypertriglyceridemic rat.](http://www.ncbi.nlm.nih.gov/pubmed/8352462) Ann N Y Acad Sci. 1993, 683:353-6.

Fickova M, Klimes J, Bohov P The effect of dietary supplementation of EPA/DHA on insulin binding to isolated rat adipocytes of hereditary hypertriglyceridemic rats on high sucrose diet. In: Klimes I, HowardBV, Kahn RC eds. Insulin and the cell membrane. New York: Harwood Academic Publishers, 1990, 429-42.

Fickova M, Sebokova E, Hubert P, Staedel C, Bohov P, Klimes J, Cremel G, Macho L. [Liver plasma membrane lipid composition and insulin receptor tyrosine kinase activity in HTG rat.](http://www.ncbi.nlm.nih.gov/pubmed/1319388) Horm Metab Res. 1992 Jan;24(1):42-3.

Klimes J, Fickova M, Vrana A. The effect of dietary supplementation of EPA/DHA on insulin binding to isolated rat adipocytes of hereditary hypertriglyceridemic rats on high sucrose diet. In: Klimes I, Howard BV, Kahn RC eds. Insulin and the cell membrane. New York: Harwood Academic Publishers, 1990, 413-27.

Klimes I, Vrána A, Kunes J, Seböková E, Dobesová Z, Stolba P, Zicha J. [Hereditary hypertriglyceridemic rat: a new animal model of metabolic alterations in hypertension.](http://www.ncbi.nlm.nih.gov/pubmed/7670646) Blood Press. 1995 4(3):137-42

Kunes J, Bin Talib HK, Dobesová Z, Vrána A, Zicha J. [Erythrocyte ion transport alterations in hypertriglyceridaemic rats.](http://www.ncbi.nlm.nih.gov/pubmed/8306545) Clin Sci (Lond). 1994, 86(1):11-3.

Lichardus B, Seböková E, Jezová D, Mitková A, Zemánková A, Földes O, Vrána A, Klimes I. [Effect of a low salt diet on blood pressure and vasoactive hormones in the hereditary hypertriglyceridemic rat.](http://www.ncbi.nlm.nih.gov/pubmed/8352450) Ann N Y Acad Sci. 1993, 683:289-94.

[Seböková E](http://www.ncbi.nlm.nih.gov/pubmed?term=Seb%C3%B6kov%C3%A1%20E%5BAuthor%5D&cauthor=true&cauthor_uid=8789319), [Klimes I](http://www.ncbi.nlm.nih.gov/pubmed?term=Klimes%20I%5BAuthor%5D&cauthor=true&cauthor_uid=8789319), [Moss R](http://www.ncbi.nlm.nih.gov/pubmed?term=Moss%20R%5BAuthor%5D&cauthor=true&cauthor_uid=8789319), [Mitková A](http://www.ncbi.nlm.nih.gov/pubmed?term=Mitkov%C3%A1%20A%5BAuthor%5D&cauthor=true&cauthor_uid=8789319), [Wiersma M](http://www.ncbi.nlm.nih.gov/pubmed?term=Wiersma%20M%5BAuthor%5D&cauthor=true&cauthor_uid=8789319), [Bohov P](http://www.ncbi.nlm.nih.gov/pubmed?term=Bohov%20P%5BAuthor%5D&cauthor=true&cauthor_uid=8789319). Decreased glucose transporter protein (GLUT4) in skeletal muscle of hypertriglyceridaemic insulin-resistant rat. [Physiol Res.](http://www.ncbi.nlm.nih.gov/pubmed?term=Klimes%20and%20GLUT4) 1995;44(2):87-92.

Seböková E, Klimes I, Moss R, Mitková A, Wiersma M, Bohov P. [Decreased glucose transporter protein (GLUT4) in skeletal muscle of hypertriglyceridaemic insulin-resistant rat.](http://www.ncbi.nlm.nih.gov/pubmed/8789319) Physiol Res. 1995;44(2):87-92.

Seböková E, Klimes I, Hermann M, Minchenko A, Mitková A, Hromadová M. [Modulation of the hypolipidemic effect of fish oil by inhibition of adipose tissue lipolysis with acipimox, a nicotinic acid analog.](http://www.ncbi.nlm.nih.gov/pubmed/8102515) Ann N Y Acad Sci. 1993, 683:183-91.

Stolba P, Opltová H, Husek P, Nedvídková J, Kunes J, Dobesová Z, Nedvídek J, Vrána A. [Adrenergic overactivity and insulin resistance in nonobese hereditary hypertriglyceridemic rats.](http://www.ncbi.nlm.nih.gov/pubmed/8394665) Ann N Y Acad Sci. 1993, 683:281-8.

Stolba P, Dobesová Z, Husek P, Opltová H, Zicha J, Vrána A, Kunes J. [The hypertriglyceridemic rat as a genetic model of hypertension and diabetes.](http://www.ncbi.nlm.nih.gov/pubmed/1513201) Life Sci. 1992, 51(10):733-40.

Vrána A, Kazdová L. [The hereditary hypertriglyceridemic nonobese rat: an experimental model of human hypertriglyceridemia.](http://www.ncbi.nlm.nih.gov/pubmed/2264160) Transplant Proc. 1990, 22(6):2579.

**Suppl. 2** *Composition of diets*

|  | **control diet** | | **high sucrose (HS)** | | **high fat (HF)** | |
| --- | --- | --- | --- | --- | --- | --- |
|  | g/kg | kJ/kg | g/kg | kJ/kg | g/kg | kJ/kg |
| casein | 100 | 1627 | 85.2 | 1342 | 85.2 | 1342 |
| dried milk | 60 | 1237 | 60 | 1237 | 60 | 1237 |
| alfalfa | 411 | 3699 | 22 | 198 | 22 | 198 |
| yeast | 60 | 256 | 60 | 256 | 60 | 256 |
| soybean oil | 6 | 222 | 6 | 222 | 6 | 222 |
| lard | - | - | - | - | 200 | 7200 |
| sucrose | - | - | 416 | 6989 | - | - |
| starch | 376 | 5109 | 100 | 1360 | 100 | 1360 |
| gelatine | - | - | 8.5 | 109 | 8.5 | 109 |
| vitamin mixture | 10 |  | 10 |  | 10 |  |
| mineral mixture | 10 |  | 10 |  | 10 |  |
| sum |  | **12150** |  | **11924** |  | **11713** |

**Suppl. 3** *Estimation of food intake*.


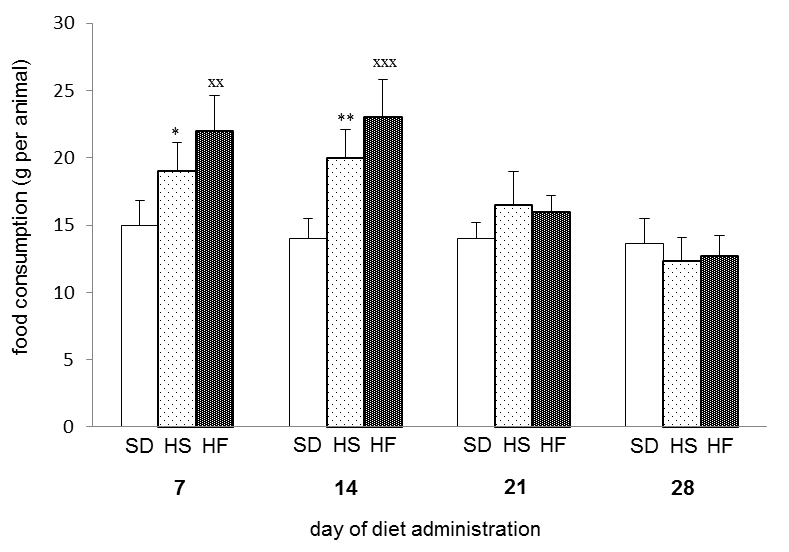


The actual amount of the diet consumed by the animals was checked once per week (at day 7, 14, 21, 28) by weighting the diet at the beginning and at the end of 24 hrs feeding period. Four animals were housed per cage, average intake per one animal was calculated as the weight difference pre- and post-feeding period divided by four.

* p < 0.05, ** p < 0.01 HS vs. SD; ^xx^ p < 0.01, ^xxx^ p < 0.001 HF vs. SD
